# Efficacy of COVID-19 mRNA vaccination in patients with autoimmune disorders: humoral and cellular immune response

**DOI:** 10.1186/s12916-023-02868-w

**Published:** 2023-06-14

**Authors:** Federica Filippini, Mauro Giacomelli, Chiara Bazzani, Micaela Fredi, Paolo Semeraro, Cesare Tomasi, Franco Franceschini, Arnaldo Caruso, Ilaria Cavazzana, Cinzia Giagulli

**Affiliations:** 1 Section of Microbiology, Department of Molecular and Translational Medicine, University of Brescia, 25123 Brescia, Italy; 2 Section of Microbiology, ASST Spedali Civili of Brescia, 25123 Brescia, Italy; 3 Rheumatology and Clinical Immunology, ASST Spedali Civili of Brescia, Brescia, Italy; 4 Rheumatology and Clinical Immunology, ASST Spedali Civili of Brescia and Department of Clinical and Experimental Sciences, University of Brescia, 25123 Brescia, Italy

**Keywords:** COVID-19 vaccination, Autoimmune diseases, Abatacept, Rituximab, T cell, Interferon-γ

## Abstract

**Background:**

The impact of immunosuppressive therapies on the efficacy of vaccines to SARS-CoV-2 is not completely clarified. We analyzed humoral and T cell-mediated response after COVID-19 mRNA vaccine in immunosuppressed patients and patients with common variable immunodeficiency disease (CVID).

**Patients:**

We enrolled 38 patients and 11 healthy sex- and age-matched controls (HC). Four patients were affected by CVID and 34 by chronic rheumatic diseases (RDs). All patients with RDs were treated by corticosteroid therapy and/or immunosuppressive treatment and/or biological drugs: 14 patients were treated with abatacept, 10 with rituximab, and 10 with tocilizumab.

**Methods:**

Total antibody titer to SARS-CoV-2 spike protein was assessed by electrochemiluminescence immunoassay, CD4 and CD4-CD8 T cell-mediated immune response was analyzed by interferon-γ (IFN-γ) release assay, the production of IFN-γ-inducible (CXCL9 and CXCL10) and innate-immunity chemokines (MCP-1, CXCL8, and CCL5) by cytometric bead array after stimulation with different spike peptides. The expression of CD40L, CD137, IL-2, IFN-γ, and IL-17 on CD4 and CD8 T cells, evaluating their activation status, after SARS-CoV-2 spike peptides stimulation, was analyzed by intracellular flow cytometry staining. Cluster analysis identified cluster 1, namely the “high immunosuppression” cluster, and cluster 2, namely the “low immunosuppression” cluster.

**Results:**

After the second dose of vaccine, only abatacept-treated patients, compared to HC, showed a reduced anti-spike antibody response (mean: 432 IU/ml ± 562 vs mean: 1479 IU/ml ± 1051: *p* = 0.0034), and an impaired T cell response, compared with HC. In particular, we found a significantly reduced release of IFN-γ from CD4 and CD4-CD8 stimulated T cells, compared with HC (*p* = 0.0016 and *p* = 0.0078, respectively), reduced production of CXCL10 and CXCL9 from stimulated CD4 (*p* = 0.0048 and *p* = 0.001) and CD4-CD8 T cells (*p* = 0.0079 and *p* = 0.0006). Multivariable General Linear Model analysis confirmed a relationship between abatacept exposure and impaired production of CXCL9, CXCL10, and IFN-γ from stimulated T cells. Cluster analysis confirms that cluster 1 (including abatacept and half of rituximab treated cases) showed a reduced IFN-γ response, as well as reduced monocyte-derived chemokines All groups of patients demonstrated the ability to generate specific CD4 T activated cells after spike proteins stimulation. After the third dose of vaccine, abatacept-treated patients acquired the ability to produce a strong antibody response, showing an anti-S titer significantly higher compared to that obtained after the second dose (*p* = 0.0047), and comparable with the anti-S titer of the other groups.

**Conclusions:**

Patients treated with abatacept showed an impaired humoral immune response to two doses of COVID-19 vaccine. The third vaccine dose has been demonstrated to be useful to induce a more robust antibody response to balance an impaired T cell-mediated one. All patients, exposed to different immunosuppressive drugs, were able to produce specific CD4-activated T cells, after spike proteins stimulation.

**Trial registration:**

Local Ethical Committee NP4187.

**Supplementary Information:**

The online version contains supplementary material available at 10.1186/s12916-023-02868-w.

## Background

Patients with autoimmune rheumatic diseases (RDs), receiving immunosuppressive therapies, are known to have an increased risk for severe bacterial and viral infections [1] and are considered clinically vulnerable to SARS-CoV-2 infection [2, 3].

To date, clinical trials related to COVID-19 vaccine excluded subjects receiving immunosuppression [4, 5], usually considered poor responders to vaccines [6, 7]. Actually, their immune response may vary accordingly with different specific immunosuppressive regimens. Patients undergoing treatment with rituximab are known to show impaired humoral responses to influenza and pneumococcal vaccination [7–10].

Up to now, data regarding COVID-19-vaccinated patients with autoimmune diseases are not conclusive. There is an urgent need to understand the impact of immunosuppressive therapies on the efficacy of vaccines to SARS-CoV-2 to obtain useful information for clinical practice. SARS-CoV-2 vaccination induces spike protein-specific antibodies with neutralizing activity [11]. Nevertheless, the duration of such humoral protection is unclear, since several studies have demonstrated waning antibody levels over time [12]. On the other hand, different authors have highlighted potent T cell-mediated responses to SARS-CoV-2 infection in COVID-19 vaccinated individuals [13, 14] with a robust long-term specific T cell responses [15, 16]. Indeed, peripheral T cell lymphopenia appeared to be correlated with increased COVID-19 severity [17], while the COVID-19 recovery is often associated with the occurrence of reactive CD4 and CD8 T cells [18]. Moreover, a recent study demonstrated that the B.1.1.7 and B.1.351 variants of concern of SARS-CoV-2 are able to partially evade humoral immunity, while CD4 T cell activation was unaffected [19]. Kalimuddin et al. also show that an early T cell response in vaccinated individuals may play a central role in generating early protection against COVID-19 [20].

All of the above-mentioned data support the need to analyze both humoral and cellular responses, in order to assess COVID-19 vaccine immunogenicity. In this study we evaluated the capability of patients, treated with abatacept (ABA), or rituximab (RTX), or tocilizumab (TCZ), as well as patients affected by common variable immunodeficiency (CVID), to generate both antibody and specific T cell-mediated responses after the second and third dose of COVID-19 mRNA-based vaccine.

## Methods

### Patients

We enrolled 40 patients, followed up by the Rheumatology and Clinical Immunology Unit, of whom 36 were affected by autoimmune RDs, 4 patients by CVID, and 12 healthy controls (HC). In order to exclude a previous SARS-CoV-2 infection, which could influence the humoral and cellular response considered in the study, we checked total antibodies directed against the SARS-CoV-2 nucleocapsid protein: 2 patients and 1 HC were consequently excluded due to serological positivity. The cohort was therefore composed of 38 consecutive adult patients (aged ≥ 18 years), followed up by the outpatients’ clinic, and 11 age-matched and comparable for sex distribution HC, vaccinated with mRNA COVID-19 vaccine (Table 1).

**Table 1:** Clinical characteristics of patients (*n*, 38) and healthy controls (*n*, 11)

	Patients *n*, 38 (%)	HC *n*, 11 (%)
Female	33 (87)	6 (55)
Age, median (IQR), years	63 (53–69)	66 (56–69)
Disease duration, median (IQR), years	14 (11–21)	
Diagnosis:		
Rheumatoid arthritis	24 (63)	
Systemic vasculitis	4 (10.5)	
Idiopathic inflammatory myositis	3 (7.9)	
Systemic lupus erythematosus	1 (2.6)	
Systemic sclerosis	2 (5.2)	
Common variable immunodeficiency	4 (10.5)	
Treatment:		
Biologic DMARDs	34/38 (89)	
ABA	14/34 (41.2)	
TCZ	10/34 (29.4)	
RTX	10/34 (29.4)	
IVIG supplementation	4/38 (11)	
Conventional DMARD-associated therapy	21/34 (44)	
Methotrexate	15/34 (44.1)	
Leflunomide	3/34 (8.8)	
Cyclosporine	2/34 (5.8)	
Mycophenolate-mofetil	1/34 (2.9)	
Glucocorticoids associated	22 (57.9)	
Daily dose, median (IQR), mg	3 (1–5)	
< 5 mg PDN	9/22 (40.9)	
≥ 5 mg PDN	13/22 (59)	

*ABA* Abatacept, *CVID* Common variable immunodeficiency, *DMARDs* Disease-modifying anti-rheumatic drugs, *IQR* Interquartile range, *IVIG* Intravenous immunoglobulin, *PDN* Prednisone, *RTX* Rituximab, *TCZ* Tocilizumab

All of them were analyzed after the second dose of vaccine. Twenty-four patients were affected by seropositive rheumatoid arthritis (RA), characterized by both RF + and ACPA + in 13 and single RF + or ACPA + in 11 cases; 4 patients were affected by CVID with IgG levels above 500 mg/dL before vaccination; 4 patients were affected by systemic vasculitis (SV): 2 of them with ANCA antibodies. Three patients were affected by idiopathic inflammatory myositis (IIM), with anti-SRP, anti-PL12 (on case each), anti-Ro52 (2 cases), anti-Ku (1 case); 2 by Systemic Sclerosis (SSc), with anti-centromere and anti-Topoisomerase I, each; 1 by Systemic Lupus Erythematosus (SLE) with ANA antibodies, without anti-dsDNA.

Clinical characteristics of the included patients and healthy controls are reported in Tables 1 and 2. All patients with RDs were treated by chronic corticosteroid therapy, immunosuppressive treatment, and/or biological drugs: in particular, 14 patients were treated with ABA, 10 with RTX, and 10 with TCZ. ABA, RTX, and TCZ are approved for severe RA and included in current recommendations for the treatment of RA [21]. Patients with CVID were treated by monthly infusion of IVIG, as immunoglobulin supplementation. No significant differences were found between ABA, RTX, TCZ, and CVID groups in terms of sex distribution, mean age, disease duration, rate of corticosteroid-treated patients, mean daily dose of steroids, and rate of DMARDs- treated patients (Table 2).

**Table 2:** Demographic and clinical characteristics of different groups: comparisons between groups resulted not significative

	ABA *n*, 14	TCZ *n*, 10	RTX *n*, 10	CVID *n*, 4
Female	13 (92.8%)	8 (80%)	8 (80%)	3 (75%)
Age, mean (SD), years	62.4 (10.7)	63 (16.3)	57.3 (15.6)	66.2 (10.8)
Disease duration, mean (SD), years	19.8 (11.7)	14.4 (4.6)	16.3 (11.9)	18.2 (10.5)
Corticosteroid associated	11 (78.6%)	6 (60%)	6 (60%)	NA
Daily dose, median (IQR), mg	3.76 (2.67)	3.8 (2.14)	4.28 (3.26)	NA
Methotrexate	7 (50%)	3 (30%)	3 (30%)	NA
Leflunomide	2 (14.3%)	1 (10%)	0	NA
Cyclosporine	0	1 (10%)	1 (10%)	NA
Mycophenolate-mofetil	0	1 (10%)	0	NA

*ABA* Abatacept, *CVID* Common variable immunodeficiency, *DMARDs* Disease-modifying anti-rheumatic drugs, *IQR* Interquartile range, *IVIG* Intravenous immunoglobulin, *PDN* Prednisone, *RTX* Rituximab, *TCZ* tocilizumab

Blood samples were collected from 6 to 8 weeks after the second dose of vaccine from June 2021 to July 2021.

Vaccinations were carried out after 4.9 weeks from the last ABA treatment, 6 months from the last RTX infusion, and 15 days from the last TCZ treatment (according to EULAR/ACR recommendations). The mean number of days between the last drug infusion and the sampling after the second vaccination was 29 for ABA, 21 for TCZ, and 251 for RTX. For controls, the mean number of days between the second vaccination and the sampling was 60.

Thirty-six patients were further analyzed after the third dose of vaccine: 2 patients out of 38 did not receive the third dose of vaccine due to refusal in one case and intercurrent death in the other. Blood samples were collected from 6 to 8 weeks after the third dose of vaccine, from December 2021 to January 2022.

## Methods

### Antibody response

The quantitative determination of total antibodies (IgA, IgM, and IgG) to the SARS-CoV-2 spike protein and nucleocapsid protein were performed by Elecsys® anti-SARS-CoV-2 electrochemiluminescence immunoassays (ECLIA; Roche Diagnostics International Ltd, Rotkreuz, Switzerland). The serum samples were run on the Roche Cobas platform according to the manufacturer’s protocol. Results were automatically calculated in U/mL in the form of a cutoff index (COI), with values < 0.80 and < 1.0 interpreted as non-reactive (negative) and ≥ 0.80 and ≥ 1.0 U/mL as reactive (positive) for anti-SARS-CoV-2 S and N antibodies, respectively.

### T cell response

The T cell response was assessed by the QuantiFERON (QFN) SARS-CoV-2 assay (Qiagen, Qiagen, Hilden, Germany), which is an interferon-γ release assay (IGRA). This test is based on in vitro CD4 or CD4 and CD8 lymphocytes stimulation in heparinized whole blood with a combination of proprietary specific SARS-CoV-2 antigens (Ag1 and Ag2) covering the S protein, followed by measurement in plasma of IFNγ production by enzyme-linked immunosorbent assay (QuantiFERONⓇ ELISA).

In particular, the QuantiFERON SARS-CoV-2 Starter Set Blood Collection Tubes consist of two tubes, named Ag1 and Ag2, which detect IFN-γ production by CD4 T cells, and CD4 and CD8 T cells together, respectively. The QFN SARS-CoV-2 Ag1 tube contains CD^+^ epitopes derived from the S1 subunit (Receptor Binding Domain) of the Spike protein, the Ag2 tube contains CD4 and CD8 epitopes from the S1 and S2 subunits of the Spike protein; the Control Tube Set contains negative (Nil) and positive (Mitogen) control tubes. The S1 subunit of the spike protein was used in order to minimize possible non-specific responses to other Coronaviruses, as observed by other authors [18]. The S2 portion of the spike protein is the most conserved region of the protein and very similar in all different Coronaviruses, thus being able to generate non-specific responses to other coronaviruses which patients may have been exposed to in the past.

All tubes were incubated at 37 °C for 16–24 h, then centrifuged for 15 min at 2500 g to harvest the plasma. Recovered plasma from the stimulated samples is used for the detection of IFN-γ using Personal LAB ELISA-based platform (Adaltis, Milano, Italy). Specimens were processed according to the manufacturer’s guidelines. The final IFN-γ values (IU/ml) were obtained by subtracting the Nil value from the raw data to avoid non-specific results. A positive response was defined for the cutoff value ≥ 0.15 IU/ml.

### Cell culture, peptide pool stimulation, and chemokine profile analysis

The whole blood was collected in BD Vacutainer Plus Plastic Whole Blood Tubes (BD Biosciences, San Jose, CA) with heparin as an anticoagulant. Peripheral Blood Mononuclear Cells (PBMCs) were obtained by Ficoll-HyPaque Lymphocyte density gradient centrifugation (Cedarlane, Ontario, Canada), washed in PBS buffer (Euroclone, Milano, Italy), and frozen at − 80 °C until use. PBMCs were plated at a density of 1 × 10^6^ cells in 96-well round bottom plates and cultured in RPMI medium supplemented with 60 mg/L penicillin, 12.5 mg/L streptomycin, and 10% heat-inactivated Fetal Bovine Serum (FBS) (Euroclone).

PBMCs were unstimulated or stimulated overnight with 1 μg/ml of peptide pool (PepTivator SARS-CoV-2 Prot_S1; Miltenyi Biotec, San Diego, CA). The pool of lyophilized peptides consists mainly of 15-mer sequences with 11 amino acids overlap, covering the N-terminal S1 domain of the glycoprotein S of SARS-CoV-2.

#### Chemokine profile levels

After peptide stimulation, plasma was recovered from QuantiFERON SARS-CoV-2 Blood Collection Tubes (Qiagen), and the evaluation of chemokines, as CXCL10 (IP-10), CXCL8 (IL-8), CXCL9 (MIG), CCL5 (RANTES), and CCL2 (MCP-1), was performed with Cytometric Bead Array method using the Human Chemokine Kit (BD Bioscience). Plasma was processed according to the manufacturer's instructions. Then, samples were acquired on BD FACSCelesta Cell Analyzer and analyzed by FCAP v3 software (BD Bioscience).

#### Intracellular cytokine staining

After 2 h from the addition of the peptide pool, PBMC were added with 1 μg/mL of brefeldin A and 1 μg/mL of Monesin (Sigma Aldrich, St. Louis, MO). After treatment, cells were collected, washed with PBS buffer (Euroclone), and stained for 10 min at 4° C with LIVE/DEAD® BD Fixable Viability Stain 700 (BD Biosciences). After a further wash in PBS buffer, cells were treated with FcR blocking reagent (Miltenyi Biotec, Milano, Italy) at 4° C for 10 min and stained with the appropriate combination of antibodies (Table S1) in 50 μl of Brilliant stain buffer (BD Biosciences). In particular, a mixture of anti-CD3, CD4, and CD8 antibodies was added to the cells for 15 min at room temperature, washed in PBS, and fixed with 100 μl of buffer A (FIX & PERM kit; Thermofisher, Waltham, MA). After washing, anti-IL-2, IL-17, IFN-γ, CD40L, and CD137 antibodies were added in 100 μl of buffer B for 20 min (FIX & PERM kit by Thermofisher), then further washed and acquired on BD FACSCelestaTM Cell Analyzer.

All data were analyzed by FlowJo software version 10.0 (Tree Star Inc., Ashland, OR) and the percentage of unstimulated cells was subtracted from the percentage of stimulated ones for each pair of stimulated and unstimulated samples.

### T cells subset analysis

Fresh whole blood (200 µl) was stained for T immunophenotype analysis, as previously described [22]. After LIVE/DEAD staining, blood samples were stained with the following combination of monoclonal antibodies (BD Bioscience): anti-CCR7, CD4, CD28, CD8, CD95, CD45RO, and CD3. Samples were then lysed and fixed in Fix and Lyse Buffer (BD Bioscience). Finally, the samples were washed and acquired on BD FACSCelesta Cell Analyzer. Data were analyzed by FlowJo software 10.0 version (Tree Star Inc.).

### Specific T cell repertoire analysis

Specific CD4 T or CD8 lymphocytes to SARS-CoV-2 spike protein were assessed by cytometry, studying the expression of early activation markers, such as CD40L and CD137, and intracellular cytokine, such as IL-2, IFN-γ, and IL-17, according to previous studies in which reactive T cells were detected through co-expression of CD137 and CD40L or between co-expression of CD40L and cytokines [18, 23].

### Statistical analysis

The sample size was calculated by Epi Info® software ver. 7, included in the study and approved by the local ethical committee: it was calculated as 37 subjects in relation to the primary objective considering a global prevalence of rheumatic diseases of 2.5%, assuming a potentially infinite population size, acceptable margin of error of 5% and confidence interval of 95%.

Data were obtained from multiple independent experiments in order to ensure reproducibility of the findings. Data are expressed as the means ± the standard deviations (SD) in the text and median with interquartile range (IQR) in the figures and tables. Student’s *t*-test and Mann–Whitney *U* test have been used to compare continuous variables of independent groups. Relationships between the continuous dependent variables Y and two groups as independent variables X were analyzed using linearly independent pairwise comparisons and Multivariable General Linear Model (GLM) analysis. *R*2 values, namely determination coefficients, are used to explain how strong the *x* variables are related to the *y* variable (shown in Additional file 1. Supplemental Table 1A).

In order to control the correlation structure among the different variables, a cluster analysis was done. Before proceeding to the cluster analysis, we verified that there was no high correlation between the variables for the dataset, because none of the values got too close to 1 or − 1. Cluster analysis was performed using the SPSS software using Ward’s method (with dendrogram) (Additional file 2. Supplemental Fig. 1), which allows the minimization of the variance within the groups and then, any distance can be used for its application.

Therefore, in choosing the distance to use, we proceeded with the quadratic Euclidean distance. We performed the analysis considering three and two clusters; two clusters were finally considered because they were well-balanced and composed of between 18 and 20 subjects. No association between the two ranks of cluster and the four groups was found.

Cluster 1 included most of Abatacept treated patients and half of the rituximab-treated patients: this cluster was named “high immunosuppression.” Cluster 2, including most tocilizumab, half rituximab, and most CVID cases, was named “low immunosuppression” (Additional file 3. Supplemental Table 2).

We performed an inferential analysis in order to find significant differences between variables considered in box plots in two identified clusters (as shown in Additional file 4. Supplemental Excel file).

A *p*-value < 0.05 was considered significant. Statistical analysis was performed using IBM-SPSS® software ver. 26.0.1 (IBM SPSS Inc. Chicago, IL). The use of the Stata® software ver. 17.0 (Stata Corporation, College Station, TX) was also considered for comparisons or implementations of test output.

## Results

### Antibody response

All 38 enrolled patients were negative for SARS-CoV-2 N protein antibodies, without differences in anti-N antibodies titers between groups (Additional file 5. Supplemental Fig. 2A).

**Fig. 1:**
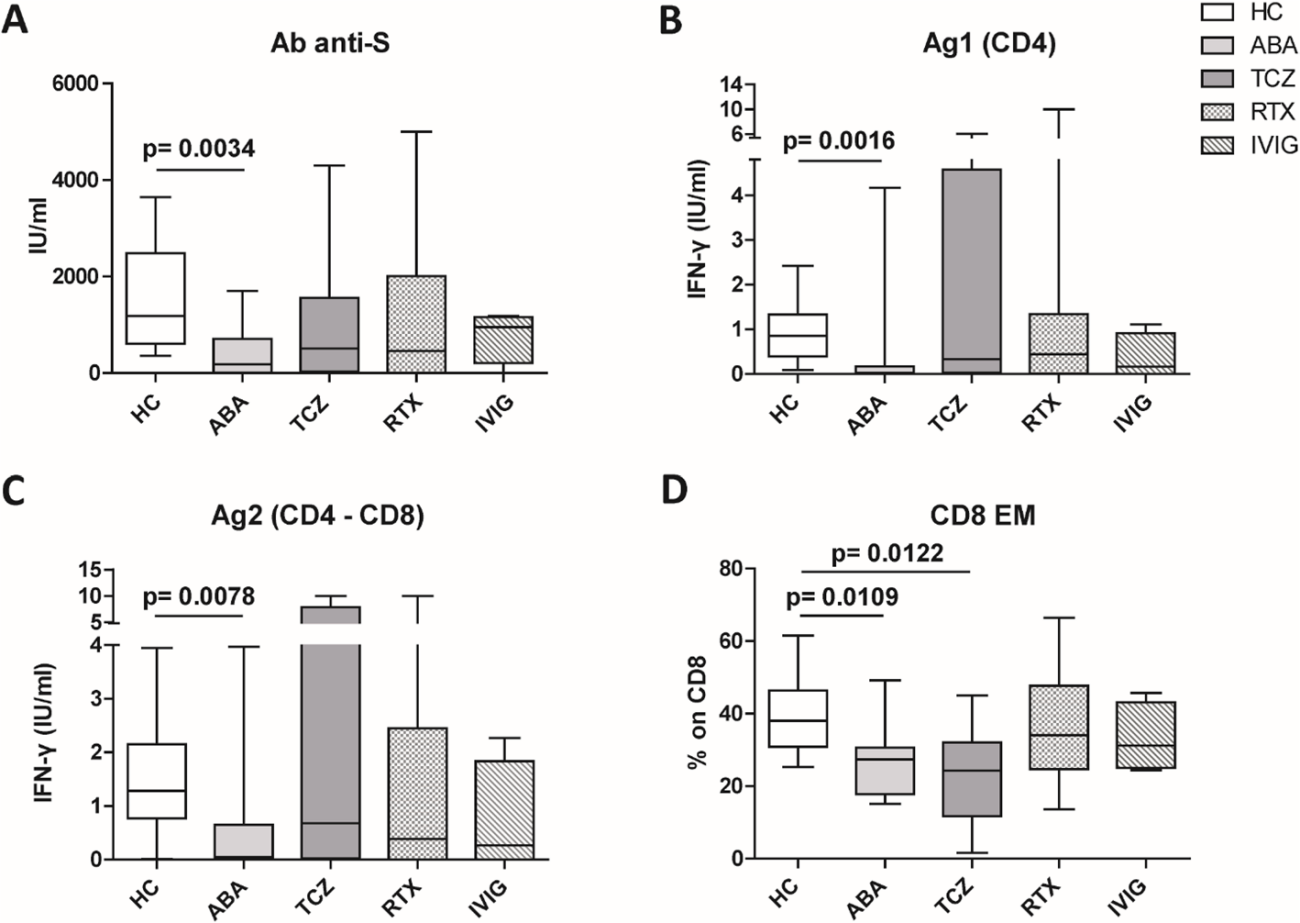
Comparison of plasma levels of antibodies anti-spike protein (**A**), IFN-γ release by T cells induced by two spike-derived peptides Ag1 (**B**) and Ag2 (**C**), effector memory CD8 T cells (**D**) among the different groups of patients, as indicated, after the second dose of COVID-19 vaccine. The data are shown as box plots (extremes of the box are at the bottom the first and at the top the third quartile, the inner row is the median, and the upper line and lower line are the highest and lowest values). HC, healthy controls; ABA, abatacept; TCZ, tocilizumab; RTX, rituximab; IVIG, intravenous immunoglobulin

The analysis of the levels of total neutralizing anti-S antibodies showed a robust antibody response to the SARS-CoV-2 S protein after the second dose of vaccine in the HC group (1479 ± 1051 IU/ml) (Fig. 1A). In TCZ-, RTX- and IVIG-treated groups the antibody titer was slightly, but not significantly, lower than HC (986 ± 1437 IU/ml, 1272 ± 1720 and 773 ± 535, respectively) (Fig. 1A). All TCZ- and IVIG-treated patients generated a humoral response, while in the RTX-treated group, the rate of seroconversion was of 70%. On the other side, ABA-treated patients showed a significantly lower antibody titer compared with HCs (432 ± 562 vs 1479 ± 1051 IU/ml, *p* = 0.0034) (Fig. 1A) and 7% of them (1/14) did not generate any anti-S antibody response. Among the different groups, only ABA-treated patients showed significantly reduced anti-S antibody levels in response to the second dose of COVID-19 vaccination, compared to HCs.

The “high immunosuppression” cluster did not show significant different anti-S response compared with the “low immunosuppression” cluster (Additional file 1. Supplemental Table 1B).

### T cell response

T cell-mediated immune response was analyzed assessing IFN-γ release by T cells induced by two different spike-derived peptides (Ag1 and Ag2).

The HC group showed a specific CD4 and CD4-CD8 T cell response with secretion of IFN-γ in response to Ag1 and Ag2 SARS-CoV-2 peptides (0.9 ± 0.66 and 1.48 ± 1.03 IU/ml, respectively), as shown in Fig. 1B and C. ABA-treated patients showed a significantly reduced IFN-γ secretion from CD4 (0.38 IU/ml ± 1.1, Fig. 1B) and CD4-CD8 stimulated T cells (0.5 ± 1.07 IU/ml, Fig. 1C) compared with HC (*p* = 0.0016 and *p* = 0.0078, respectively). The ABA-treated group also showed a reduced, but not significant, CD4 T response also compared with the TCZ-treated group (1.88 ± 2.44 IU/ml; *p* = 0.066) (Fig. 1B). Indeed, only 29% of the total ABA-treated patients demonstrated the capability of developing a T cell response to SARS-CoV-2 peptides. On the other side, the TCZ-, RTX-, and IVIG-treated subjects showed a comparable T cell response to HCs.

Overall, these data highlighted the reduced capability of ABA-treated patients to activate a T cell response with release of IFN-γ in response to SARS-CoV-2 peptide stimulation, when compared to HC. Cluster analysis confirmed these data: the “high immunosuppression” cluster, including most of ABA treated and half of RTX treated patients, showed significantly reduced IFN-γ response compared with the “low immunosuppression” cluster (Additional file 1. Supplemental Table 1B).

### T cells subpopulations

The rate of CD4 and CD8 lymphocytes and the amount of naïve, central memory, terminal differentiated, and stem memory T cells (calculated as percentages of parental cells) did not significantly vary between different groups at baseline.

By contrast, a significant reduction of CD8 effector memory T cell (CD8 Tem) subset was observed in both ABA-treated (27.41% ± 10.23%) and TCZ-treated groups (22.93% ± 13.70%), when compared with HC (38.77% ± 10.72; *p* = 0.0109 and *p* = 0.0122, respectively) (Fig. 1D). Moreover, in ABA-treated groups, we also observed a lower rate of CD4 Tem subset compared with HC without significant difference (*p* = 0.1468) (Additional file 5: Supplemental Fig. 2B), while no difference was observed in CD4/CD8 ratio and CD3 rate among the different cohorts (Additional file 5. Supplemental Fig. 2C and D). The same data was obtained using cluster analysis.

In conclusion, T cell subset analysis highlighted a significant reduction of CD8 Tem cells in ABA- and TCZ-treated patients compared with HCs.

### Chemokine profile analysis

In order to extensively analyze the T cell response after COVID-19 vaccination we assessed on the same sample of QuantiFERON assay, the main chemokines produced in response to stimulation with Ag1 and Ag2 spike-derived peptides: MIG (CXCL9) and IP-10 (CXCL10), induced by IFN-γ; MCP-1 (CCL2) and IL-8 (CXCL8), namely monocyte-derived chemokines; and CCL5 (RANTES) as chemokine involved in an inflammatory response.

**Fig. 2:**
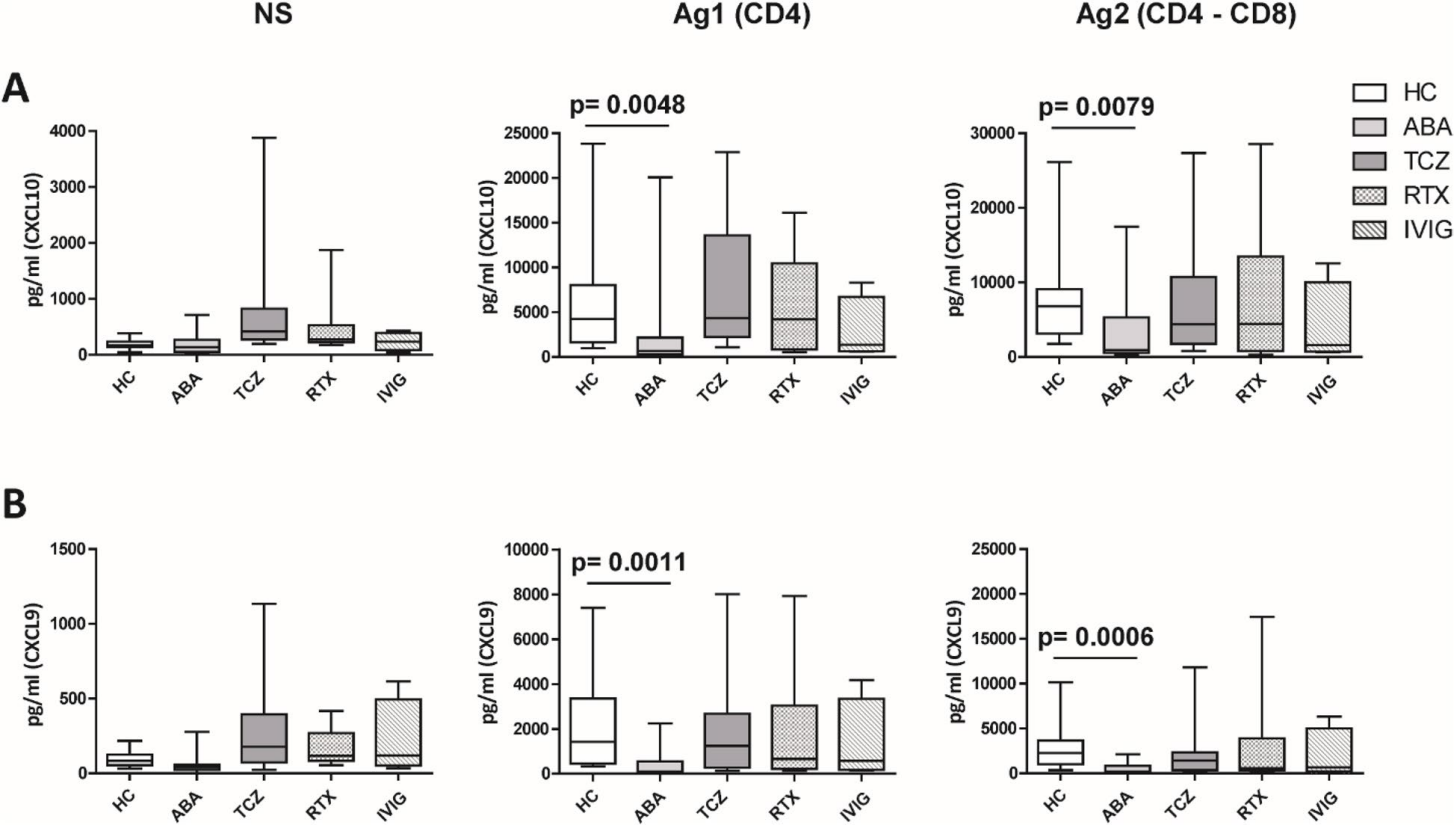
Levels of CXCL10 (**A**) and CXCL9 (**B**) released by unstimulated cells (on the left), CD4 T cells stimulated with spike-derived peptide QFN Ag1 (in the middle), and CD4/CD8 T cells stimulated with spike-derived peptide QFN Ag2 (on the right), among the different groups of patients, as indicated, after the second dose of COVID-19 vaccine. The data are shown as box plots (extremes of the box are at the bottom the first and at the top the third quartile, the inner row is the median, and the upper line and lower line are the highest and lowest values). HC, healthy controls; ABA, abatacept; TCZ, tocilizumab; RTX, rituximab; IVIG, intravenous immunoglobulin

ABA-treated subjects showed lower plasma levels of CXCL10 compared with HC, either in Ag1-stimulated (2687 ± 5333 pg/ml vs 6454 ± 6649 pg/ml, *p* = 0.0048) or in Ag2-stimulated samples (3273 ± 4830 pg/ml vs 8466 ± 7311 pg/ml, *p* = 0.0079) (Fig. 2A). Regarding CXCL9 production, we detected significantly lower levels in ABA-treated subjects than HC in both stimulation conditions with Ag1 (403.0 ± 650.1 pg/ml vs 2182 ± 2132 pg/ml, *p* = 0.0011) and Ag2 (532.8 ± 714.9 pg/ml vs 2989 ± 2665 pg/ml, *p* = 0.0006) (Fig. 2B).

The same associations were obtained taking into account cluster analysis: the “high immunosuppression” cluster showed significantly lower production of CXCL10 and CXCL9, either in Ag1-stimulated or in Ag2-stimulated samples, compared with the “low immunosuppression” cluster.

MCP-1 (CCL2) and IL-8 (CXCL8) plasma levels did not significantly vary among the different groups (Additional file 6. Supplemental Fig. 3A and 3B): a lower production of MCP-1 in ABA-treated patients was observed compared with HC, even if not statistically significant, in both stimulation conditions with Ag1 (*p* = 0.106) and Ag2 (*p* = 0.1469). Conversely, cluster analysis demonstrated that the “high immunosuppression” cluster was associated with a significantly lower production of MCP-1 and IL-8, in Ag1 stimulation (*p* < 0.0001 and *p* < 0.0001) and Ag2 stimulation regimen (*p* < 0.0001 and *p* < 0.0001), compared with the “low immunosuppression” cluster (Additional file 1. Supplemental Table 1B).

Finally, RANTES (CCL5) levels were significantly lower in ABA-treated patients than HC, only in unstimulated cells (16,720 ± 6901 pg/ml vs 22,680 ± 7652 pg/ml, *p* = 0.020) and in Ag1 stimulation regimen (20,950 ± 7413 pg/ml vs 27,660 ± 8502 pg/ml, *p* = 0.0401) (Additional file 6. Supplemental Fig. 3C). These data were not confirmed by cluster analysis.

Multivariable GLM analysis confirmed a direct relationship between ABA treatment and impaired CXCL10 in Ag2-stimulated T cells (*p* = 0.044), impaired CXCL9 in Ag1 (*p*: 0.007), and Ag2-stimulated T cells (*p*: 0.003), respectively, and reduced IFN-γ after Ag2 stimulation (*p*: 0.031) (Additional file 1. Supplemental Table 1A). The reduction of CXCL9 after both Ag1 and Ag2 stimulations is the best item associated with abatacept treatment, as demonstrated by *R*^2^ of 0.294 and 0.245, respectively.

### Fold increase of chemokines production between non-stimulated and Ag1 or Ag2 stimulated T cells

**Fig. 3:**
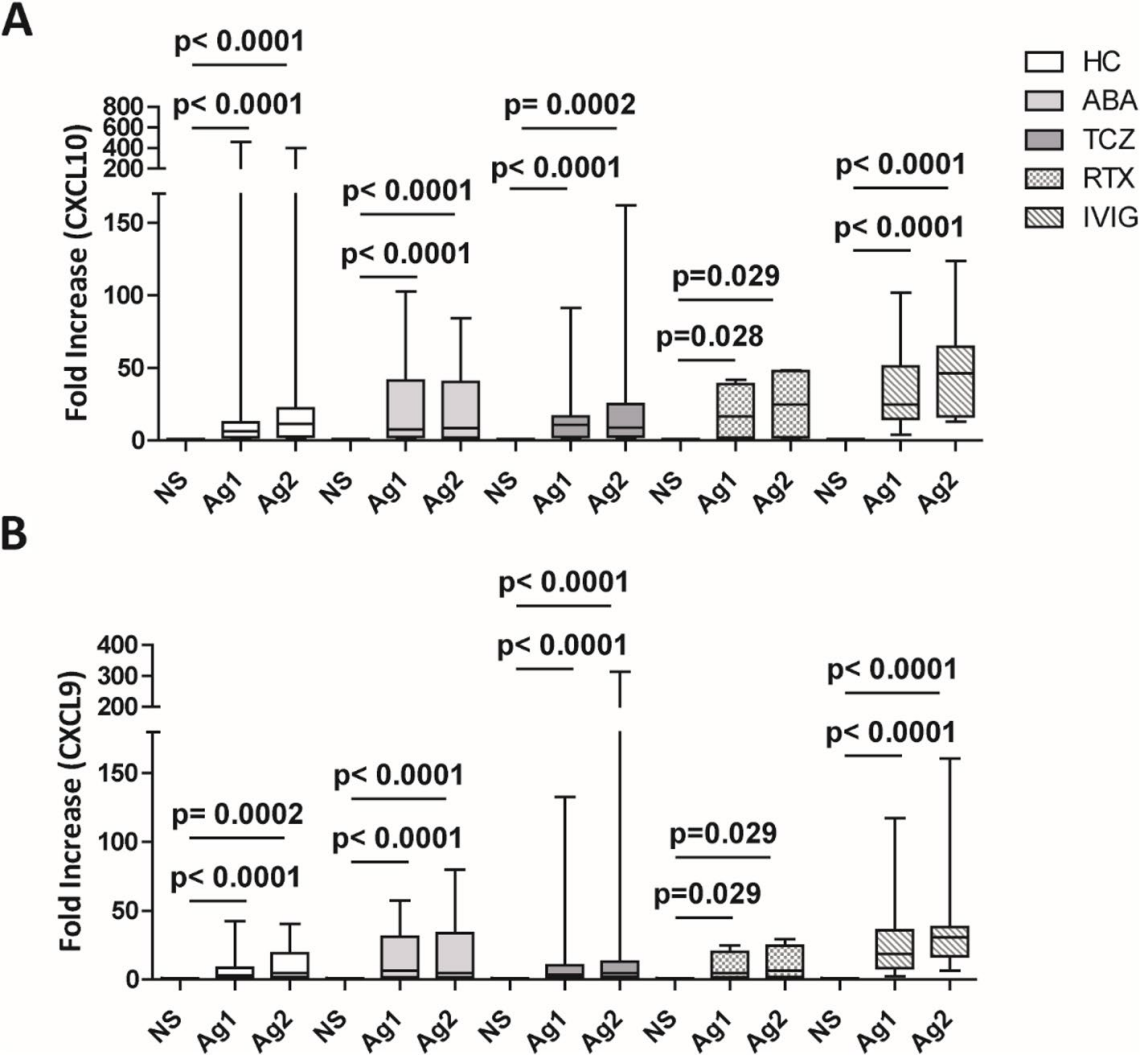
Fold increase levels of CXCL10 (**A**) and CXCL9 (**B**) between unstimulated (NS) and CD4 T cells stimulated with spike-derived peptide QFN Ag1, fold increase levels between unstimulated (NS) and CD4/CD8 T cells stimulated with spike-derived peptide QFN Ag2 for each group of patients, as indicated, after the second dose of COVID-19 vaccine. The data are shown as box plots (extremes of the box are at the bottom the first and at the top the third quartile, the inner row is the median, and the upper line and lower line are the highest and lowest values). HC, healthy controls; ABA, abatacept; TCZ, tocilizumab; RTX, rituximab; IVIG, intravenous immunoglobulin

The fold increase of chemokines levels between unstimulated and stimulated T cells with Ag1 or Ag2 was assessed in all groups (Fig. 3 and Additional file 7. Supplemental Fig. 4).

All treatment groups, as well as HC, showed a significant fold increase of CXCL10 and CXCL9 between unstimulated and Ag1-stimulated T cells, as well as between unstimulated and Ag2-stimulated T cells (Fig. 3). A significant fold increase between unstimulated and Ag1 stimulated as well as unstimulated and Ag2 stimulated T cells was observed almost in all groups also for CCL2, CXCL8, and CCL5 (Additional file 7. Supplemental Fig. 4).

Compared with HC, ABA treatment is associated to the lowest fold increase of CXCL10 and CXCL9 after Ag1 and Ag2 stimulation, compared with the other groups (Additional file 8. Supplemental Table 3).

To summarize, even if ABA-treated patients showed a reduced production of IFN-γ-inducible chemokines, as CXCL9 and CXCL10, they maintained the capability to increase the production of these chemokines, when stimulated with spike-derived peptides Ag1 and Ag2.

### Specific T cell repertoire analysis

**Fig. 4:**
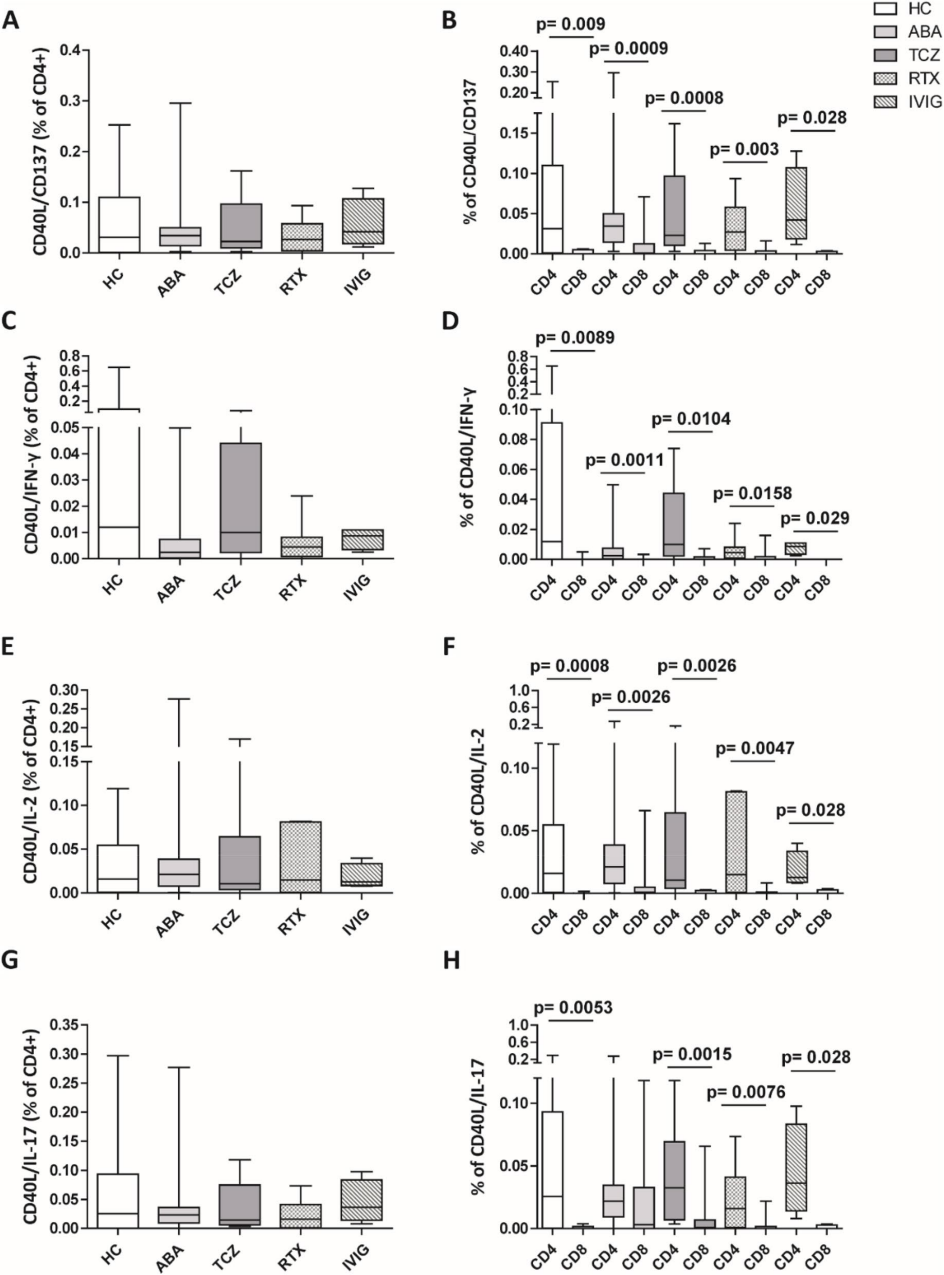
Comparison of CD4 CD40L/CD137 (**A**), CD40L/IFN-γ (**C**), CD40L/IL-2 (**E**), and CD40L/IL-17 (**G**) cell percentages between the different treatment groups of patients, after the second dose of COVID-19 vaccine. Percentage of CD40L/CD137 (**B**), CD40L/IFN-γ (**D**), CD40L/IL-2 (**F**), and CD40L/IL-17 (**H**) in CD4 and CD8 subset for each group of patients. The percentages of CD40L/CD137, CD40L/IL-2, CD40L/IFN-γ, and CD40L/IL-17 cells are calculated respect to CD4 and CD8 total lymphocytes. CD4 and CD8 double positive of unstimulated cells were subtracted from the stimulated ones. The data are shown as box plots (extremes of the box are at the bottom the first and at the top the fourth quartile, the inner row is the median, and the upper line and lower line are the highest and lowest values). HC, healthy controls; ABA, abatacept; TCZ, tocilizumab; RTX, rituximab; IVIG, intravenous immunoglobulin

Finally, we evaluated, on PBMC stimulated with specific S1 peptide, the expression of CD137, IL-2, IFN-γ, and IL-17 on CD4 and CD8 T cells in different groups of patients and in HCs. This was to assess if, after vaccination, clones of specific anti-spike T lymphocytes could have been generated. No significant difference was observed in the rate of CD40L/CD137, CD40L/IL-2, CD40L/IFN-γ, and CD40L/IL-17 double-positive T cells in different groups (Fig. 4A, C, E, G) and in two different clusters (Additional file 1. Supplemental Table 1B). Moreover, the analysis of the rates of CD4 and CD8 in CD40L/CD137, CD40L/IL-2, CD40L/IFN-γ, and CD40L/IL-17 double-positive populations showed a predominance of CD4 subset in all groups, while the activated CD8 subset was very low or even absent (Fig. 4B, D, F, H).

To conclude, COVID-19 mRNA vaccination could induce only CD4 T cells specific for SARS-CoV-2 spike protein in all treated patients as well as in HC.

### Effect of the third dose of COVID-19 mRNA vaccine

All 36 patients, analyzed after the third dose of vaccine, did not show anti-nucleocapside protein (anti-N) antibodies (Additional file 9. Supplemental Fig. 5A).

**Fig. 5:**
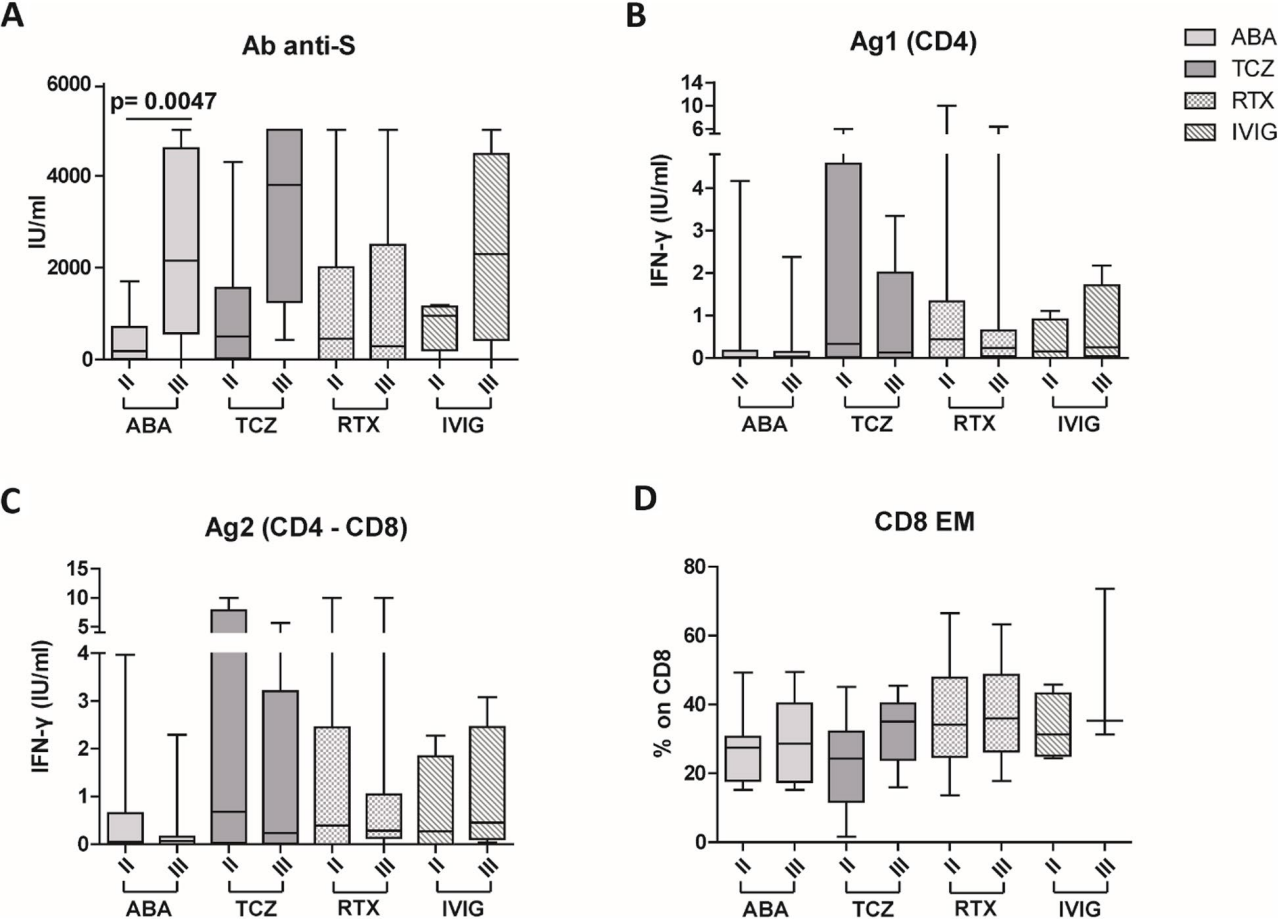
Comparison of plasma levels of antibodies anti-spike protein (**A**), IFN-γ release by T cells induced by two spike-derived peptides Ag1 (**B**) and Ag2 (**C**), effector memory CD8 (**D**) for each group of patients, as indicated, between the second and the third dose of COVID-19 vaccine. The data are shown as box plots (extremes of the box are at the bottom the first and at the top the third quartile, the inner row is the median, and the upper line and lower line are the highest and lowest value). HC, healthy controls; ABA, abatacept; TCZ, tocilizumab; RTX, rituximab; IVIG, intravenous immunoglobulin

After the third dose of the vaccine, we observed a marked and significant increase of anti-S antibodies in ABA-treated patients, compared with the antibody titer after the second dose (432.3 ± 562.0 vs 2423 ± 1896 IU/ml, *p* = 0.0047). Also, TCZ-treated and IVIG-treated groups showed an increase of antibody titer after the third dose of vaccine, but not statistically significant. By contrast, the titer of anti-S antibodies did not vary in RTX-treated patients between the second and third dose (Fig. 5A).

In all the groups of treatment, we did not observe any difference in IFN-γ release by T cells after the second and third dose of vaccine in response to Ag1 and Ag2 stimulations, respectively (Fig. 5B and C).

In addition, no difference in effector memory CD4 T cells, CD4/CD8 ratio, and CD3 percentage was observed between the second and third dose of vaccine in all groups (Additional file 9. Supplemental Fig. 5B, C, D). Likewise, the rate of effector memory CD8 T cells did not vary between the second and third dose in all groups (Fig. 5D).

Overall, these data show that ABA-treated patients, after the third dose of COVID-19 vaccine, acquired the capability to produce a strong antibody response. However, they did not recover the ability to activate a specific T cell response in response to SARS-CoV-2 peptide stimulation.

## Discussion

In this study we explored the ability of patients with autoimmune RDs, treated by different immunosuppressive therapies and CVID, treated with IVIG, to generate both humoral and specific T cell-mediated response, after two and three doses of COVID-19 mRNA vaccination. Cluster analysis identified two main classes of immunosuppression state, represented by the “high immunosuppression” cluster containing most of ABA treated patients and half of the RTX-treated cases; and the “low immunosuppression” cluster with most of TCZ, CVID patients, half of RTX, and a minority of ABA treated cases. The analysis confirmed the independence of a large amount of different variables, which resulted in significant association with the “high immunosuppression” cluster.

The main findings of the research were (i) a reduced anti-S response in the ABA-treated group, restored after the third dose of vaccine; (ii) an impaired T cell activation, represented by a reduction of IFN-γ and related chemokines; (iii) a reduction of effector memory CD8 T cells in ABA-treated group and in cluster 1 patients; and (iv) a significant ability of ABA treated group to mount a CD4 T cell response, when stimulated with spike derived antigens.

Among different immunosuppressive treatments, ABA was significantly associated with a reduced capacity to produce anti-SARS-CoV-2 spike protein antibodies, while RTX and TCZ treated-subjects displayed a slightly decreased antibody titer compared with HCs, but with a rate of seroconversion equal to 70 and 100%, respectively. These data are in line with the results of seroconversion obtained from different reports, where an excellent rate of antibody response (> 90%) in TCZ-treated patients and an attenuated rate of seroconversion (< 70%) in patients receiving ABA therapy were assessed [24–26]. Different authors reported a low rate of seroconversion in RTX-treated patients [25, 26], while in our hands this drug did not significantly influence the humoral response, that result is comparable to HCs. This discrepant data could be due to the timepoint of vaccination and sampling, considered critical for antibody response during RTX treatment [25, 26]: we performed vaccination and sampling after 6 months and 8 months after the last RTX infusion, respectively. Other groups did not report if RTX had been discontinued or delayed before vaccination and sampling procedures [25, 26], anyway the timepoint between RTX exposure and sampling seems to be shorter than that scheduled in our cohort. The third dose of COVID-19 mRNA vaccine significantly improved the humoral immune response of ABA- and TCZ-treated patients, as demonstrated by the increase of specific anti-S antibodies. These data confirmed a recent paper reporting a significant increase of anti-S titer in 4 ABA-treated patients after the third dose of vaccine [27]. By contrast, RTX failed to induce a significant anti-S antibody titer, confirming other published data [28].

Patients treated with ABA displayed, after two doses of COVID-19 mRNA vaccine, an impaired CD4 and CD4/CD8 T cell response with a reduced release of IFN-γ, as well as IFN-γ-inducible chemokines, such as CXCL9 and CXCL10, in response to peptides of the spike protein. Multivariable GLM analysis confirmed a direct relationship between ABA exposure and reduced production of CXCL9, CXCL10, and IFN-γ. This compromised response of T cells remains unvaried even after the third dose of vaccine. These results, as expected, are in line with the biological effects of ABA. Indeed, ABA, an analog of T lymphocyte-associated antigen-4 (CTLA4) expressed on activated T lymphocytes, behaves as a negative immunomodulating molecule, that inhibits the specific full activation of T lymphocytes [29, 30], leading to reduced survival of T lymphocytes, clonal expansion, cytokine production and decreased cooperation with B lymphocytes [31–35]. These biological effects can induce an altered B cell selection and compromised B cell immune response, as demonstrated in our study and other reports [25, 26].

Moreover, our data showing an impaired release of IFN-γ and production of IFN-γ-inducible chemokines are in line with previous studies, which reveal that ABA treatment could directly affect IFN-γ and CXCL10 cytokine levels [36–38]. Since IFN-γ is known to be a potent inducer of CXCL10 [38], the IFN-γ inhibition by ABA may induce a decreased production of IFN-γ related chemokines, with a possible involvement of other inflammatory cytokines, as IL-1β, and TNF [39–41]. Cluster analysis confirmed that the “high immunosuppression” cluster, including most ABA and half of RTX patients, showed a significantly lower release of IFN-γ and production of IFN-γ-inducible chemokines after spike peptides’ stimulation.

In addition, monocytes’ derived chemokines, such as MCP-1 (CCL2) and IL-8 (CXCL8), are significantly lower after spikes’ peptides stimulation. These data could be explained by the direct effect of ABA on CD80/86 of monocytes affecting their functional state and their ability to produce proinflammatory cytokines, such as TNF-α, IL-1β, IL-6, CXCL8, and CCL2 [42, 43]. Its action in the control of inflammation appears to be based on its “immunoblock” function of the monocyte effector pathways leading to proinflammatory cytokine production [43].

Published data on T cell specific response to COVID-19 vaccination demonstrated a robust and stable spike-specific CD8 T cell response in healthy subjects. In particular, high levels of specific effector memory CD8 T cells were rapidly found after exposure to different COVID-19 vaccines [44, 45]: we did not find the same data in HC, maybe due to a different timing of sampling in our work, which is longer compared to others [44, 45]. In any case, in our study, ABA- and TCZ-treated patients showed a significantly reduced rate of effector memory CD8 T cells: these data probably reflect the well-known effect of ABA on T cell side in RA patients [46], as well as the known reduction of both naive T cell and memory CD8 cells described during TCZ treatment in RA patients [47].

By contrast, in our hands the RTX-treated group did not show a significant difference in generating memory CD8 T response to vaccination compared to HC: this data is in line with the preserved repertoire of CD8 T cells subpopulations observed during RTX therapy, described by other authors, that analyzed 11 RTX- treated patients with a comparable duration of RTX withdrawal before the first dose of vaccination [48].

Despite ABA-treated patients showing an impaired humoral immune response and chemokines production, they maintained the capability to generate a CD4 T cell repertoire specific for SARS-CoV-2 spike proteins, without any difference compared with other analyzed groups. All the patients and HCs demonstrated higher levels of specific CD4 activated (namely, CD40L/CD137, CD40L/IL-2, CD40L/IFN-γ, CD40L/IL-17 double positive), compared with activated CD8 subsets levels. These data are not in line with other authors, who have highlighted a significant and rapid increase of CD8 T cells in response to COVID-19 vaccine [45]. However, the mobilization of CD8 T lymphocytes seems to represent a very early stage of the immune response to COVID-19 vaccination (within 1 or 2 weeks) [44, 45], followed by a robust CD4 T and B cells increase [44]. The high levels of activated CD4 T cells and lower CD8 could reflect a snapshot of the later timepoint of sampling, compared to what was scheduled by other groups.

Our work was limited by the low number of patients enrolled: the limited sample size did not allow generalized conclusions, because it could be considered not representative of the rheumatological population. Anyway, the enrolled cohort is composed by all the consecutive patients evaluated in our outpatients’ clinic before and after the vaccination doses, with the correct period of biological drugs’ withdrawal. As reported in the “Method” section, no other filters have been applied. So this consecutiveness warrants that the sample size is representative of all patients treated with biologics or with IVIG, belonging to our outpatients’ clinic.

This sample size is justified by the choice to have an accurate coordination between the timing of biological drugs’ withdrawal, first and second vaccine dose inoculations, and the strict timing of sampling.

This sample size is comparable to other papers published by different groups [25, 26, 45, 48], including a recent case–control study [49], all demonstrating a reduced cellular response by Aba-treated patients and reduced seroconversion rate in RTX-treated patients. No pilot studies have been performed either by us or other authors, considering the single groups of immunosuppressed patients: according to biostatistical methods, pilot studies were not performed with limited numbers of cases, because this does not allow to make inferences between data. Although preliminary, much data is accumulating from different groups [25, 26, 48, 49] identifying Aba as a negative predictor of response to SARS-CoV2 vaccination, confirming our data. Wider and multicenter studies should be advisable to overcome the limitation of the small sample size.

This bias could be partially overcome by the performance of independent experiments providing reproducible data and by different statistical methods all demonstrating a direct relationship between ABA/RTX exposition and IFN-related immune response and monocytes’ activation.

In immunosuppressed patients, vaccine-induced cellular immunity represents a good surrogate for protection [50–52]. Indeed, cellular immunity is known to play a crucial role in SARS-Cov2 infection, as specific CD4 and CD8 responses have been identified in healthy COVID-19-recovered individuals, as well as in recovered agammaglobulinemic patients [53, 54]. Performing extended cellular assessments, our work contributed to explain which kind of immune response patients chronically exposed to different immunosuppressive regimens are able to generate in response to COVID-19 vaccination. The present paper did not explore the induction of autoimmunity after COVID-19 vaccination, as well as the assessment of the B lymphocyte side of the immune response or the variation of the original autoantibody profile. This intriguing aspect could be an item for future analysis.

## Conclusion

High immunosuppression regiment leads to an impaired humoral immune response and CD8 T response to two doses of COVID-19 vaccine, anyway the ability to generate clones of CD4 T lymphocytes specific for SARS-CoV-2 spike proteins is preserved. After the third dose of COVID-19 mRNA vaccine, Abatacept-treated patients acquired the capability to produce a strong antibody response, despite this they maintained a significant reduction of CD8 T response. All these data represent a critical message from the laboratory research bench to the clinical patients’ side, suggesting that repeated vaccine doses may be necessary to optimize the immunological response and to induce more robust serological responses in these high-risk vulnerable patients.

## Supplementary Information

**Additional file 1: Supplemental table 1a.** comparison between ABA-treated groups and healthy controls in CXCL10 levels, CXCL9 levels, IFN-γ levels, after Ag1 stimulation and Ag2 stimulation, analysed by multivariable General Linear Model. **Supplemental table 1b.** comparison between “high immunosuppression” cluster and “low immunosuppression” cluster of all items analysed, as showed in box-plots.

**Additional file 2: Supplemental figure 1.** Dendogram using Ward Linkage.

**Additional file 3: Supplemental table 2.** cluster 1 and 2 composition.

**Additional file 4: Supplemental excel data.** Cluster analysis raw data.

**Additional file 5: Supplemental figure 2.** Comparison of plasma levels of antibodies anti-nucleocapsid protein (A), effector memory CD4 T cells (B), CD4/CD8 ratio balance (C) and CD3 T cells (D) among the different treatment groups of patients after the second dose of vaccine. The data are shown as box plots. HC=Healthy Controls, ABA=abatacept, TCZ=tocilizumab, RTX=rituximab, IVIG= intravenous immunoglobulin.

**Additional file 6: Supplemental figure 3.** Levels of CCL2 (A), CCL8 (B), CCL5 (C) released by unstimulated cells (on the left), CD4 T cells stimulated with spike-derived peptide the different groups of patients, as indicated, after the second dose of COVID-19 vaccine. The data are shown as box plots (extremes of the box are at the bottom the first and at the top the third quartile, the inner row is the median and the upper line and lower line are the highest and lowest values). HC=Healthy Controls, ABA=abatacept, TCZ=tocilizumab, RTX=rituximab, IVIG=intravenous immunoglobulin.

**Additional file 7: Supplemental figure 4.** Fold increase levels of CCL2 (A), CXCL8 (B) and CCL5 between unstimulated (NS) and CD4 T cells stimulated with spike-derived peptide QFN Ag1, fold increase levels between unstimulated (NS) and CD4/CD8 T cells stimulated with spike-derived peptide QFN Ag2among different groups of patients, as indicated, after the second dose ofCOVID-19 vaccine. The data are shown as box plot (extremes of the box are at the bottom the first and at the top the third quartile, the inner row is the median and the upper line and lower line are the highest and lowest values). HC=Healthy Controls, ABA=abatacept, TCZ=tocilizumab, RTX=rituximab, IVIG=intravenous immunoglobulin.

**Additional file 8: Supplemental table 3.** CXCL10 and CXCL9 fold increase (between Ag1 stimulated and non stimulated cells, and Ag2 stimulated and non stimulated cells) were higher in abatacept groups compared to healthy controls. No significant difference was found in CXCL10 and CXCL9 fold increase between other groups and healthy controls.

**Additional file 9: Supplemental figure 5.** Comparison of plasma levels of antibodies anti-nucleocapsid protein (A), effector memory CD4 T cells (B), CD4/CD8 ratio balance (C) and CD3 T cells(D) for each group of patients between the second and third dose of COVID-19 vaccine. The data are shown as box plots (extremes of the box are at the bottom the first and at the top the third quartile, the inner row is the median and the upper line and lower line are the highest and lowest value). HC=Healthy Controls, ABA=abatacept, TCZ=tocilizumab, RTX=rituximab, IVIG=intravenous immunoglobulin.

## Data Availability

The datasets used and/or analyzed during the current study are available from the corresponding author on reasonable request.

## Abbreviations

ABA: Abatacept
ACPA: Anti-citrullinated peptide antibodies
ACR/EULAR: American College of Rheumatology/European League Against Rheumatism
CVID: Common variable immunodeficiency
DMARDs: Disease-modifying anti-rheumatic drugs
GLM: General Linear Model
HC: Healthy control
IFN-γ: Interferon-γ
IIM: Idiopathic inflammatory myositis
IQR: Interquartile range
IVIG: Intravenous immunoglobulin
RA: Rheumatoid arthritis
RDs: Rheumatic diseases
RF: Rheumatoid factor
RTX: Rituximab
SD: Standard deviation
SLE: Systemic lupus erythematosus
SSc: Systemic sclerosis
SV: Systemic vasculitis
TCZ: Tocilizumab
