# Supplementary material for: Efficacy of COVID-19 mRNA vaccination in patients with autoimmune disorders: humoral and cellular immune response
Source: BMC Med. 2023 Jun 14;21:210. doi: 10.1186/s12916-023-02868-w (PMC10266318; doi:10.1186/s12916-023-02868-w)
Supplement: Supplementary file 1 — Additional file 1: Supplemental table 1a. comparison between ABA-treated groups and healthy controls in CXCL10 levels, CXCL9 levels, IFN-γ levels, after Ag1 stimulation and Ag2 stimulation, analysed by multivariable General Linear Model. Supplemental table 1b. comparison between “high immunosuppression” cluster and “low immunosuppression” cluster of all items analysed, as showed in box-plots. [file 12916_2023_2868_MOESM1_ESM.doc]

Supplemental table 1a: comparison between ABA-treated groups and healthy controls in CXCL10 levels, CXCL9 levels, IFN-γ levels, after Ag1 stimulation and Ag2 stimulation, analysed by multivariable General Linear Model.

|  | ABA group  (n.14) | HC  (n.11) | p multivariable | R² |
| --- | --- | --- | --- | --- |
| CXCL10 levels after Ag1 stimulation, median (IQR) | 646.04  (132.9-20055.58) | 4227,66  (975,5-23796,11) | 0.129 | 0.058 |
| CXCL10 levels after Ag2 stimulation, median (IQR) | 919.64 (186,32 -17450,19) | | 6780,87 | | --- |   (1770,99-26102,46) | 0.044 | 0.129 |
| CXCL9 levels after Ag1 stimulation, median (IQR) | 92,43  (22,99-2239,84) | 1420,36  (331,16-7396,36) | 0.007 | 0.245 |
| CXCL9 levels after Ag2 stimulation, median (IQR) | 176,13  (24,21-2138,90) | 2258,80  (354,3-10128,63) | 0.003 | 0.294 |
| IFN-γ levels after Ag1 stimulation, median (IQR) | 0,02  (0-4.17) | 0,85 (0,09-2,42) | 0.183 | 0.036 |
| IFN-γ levels, after Ag2 stimulation, median (IQR) | 0,05  (0-3.97) | 1,28  (0,01-3,95) | 0.031 | 0.152 |

Supplemental table 1B: comparison between “high immunosuppression” cluster and “low immunosuppression” cluster of all items analysed and showed in box-plots

|  | High immunosuppression | Low immunosuppression | p |
| --- | --- | --- | --- |
| CD3 (% on Lympho), median (IQR) | 55.3 (22.8-79) | 60.95 (34.4-83.6) | 0.633 |
| CD4/CD8, median (IQR) | 1.84 (0.4-4.2) | 2.03 (0.53-3.76) | 0.534 |
| CD4 EM (on %CD4), median (IQR) | 25.7 (1.6-49.2) | 31.1 (15.6-66.5) | 0.051 |
| CD4 EM (on %CD4), median (IQR) | 27.85 (5.74-58.2) | 28.9 (14.3-74.8) | 0.276 |
| IFN-γ levels after Ag1 stimulation, median (IQR) | 0.02 (0.00-0.46) | 0.53 (0.01-11) | <0.0001 |
| IFN-γ levels, after Ag2 stimulation, median (IQR) | 0.035 (0.00-0.74) | 10.2 (0.00-11) | 0.005 |
| Anti-S ab | 254.3 (0.4-5001.0) | 505.2 (0.4-3801) | 0.718 |
| MCP-1 non stimulated levels, median (IQR) | 817.97 (166.16-4816.13) | 3577.40 (832.95-24670.33) | <0.0001 |
| MCP-1 after Ag1 stimulation, median (IQR) | 2412.54 (657.95-7755.068) | 11046.86 (2367.49-109045.53) | <0.0001 |
| MCP-1 after Ag1 stimulation, median (IQR) | 4132.31 (397-7581.3) | 13628.9 (1935.54-119212.79) | <0.0001 |
| CXCL10 non stimulated, median (IQR) | 227.72 (27.7-3881.47) | 261.18 (43.8-1061) | 0.593 |
| CXCL10 levels after Ag1 stimulation, median (IQR) | 888.06 (132.9-6808.66) | 4539.26 (508-51-22883.75) | 0.001 |
| CXCL10 levels after Ag2 stimulation, median (IQR) | 972.50 (186.3-9855) | 5699.84 (364.35-28540.20) | 0.010 |
| CXCL9 non stimulated, median (IQR) | 75.84 (0.83-1132.8) | 141.68 (24.71-614.46) | 0.067 |
| CXCL9 levels after Ag1 stimulation, median (IQR) | 141.79 (17.22-1245.5) | 1335.12 (81.64-8010.87) | <0.0001 |
| CXCL9 levels after Ag2 stimulation, median (IQR) | 191.83 (24.2-1571.68) | 1617.040 (49.29-17426.11) | <0.0001 |
| IL-8 non stimulated levels, median (IQR) | 7978, 273 (1575.86-30866.21) | 36344.94 (11589.02-84486.38) | <0.0001 |
| IL-8 levels after Ag1 stimulation, median (IQR) | 19320.65 (2260.81-37583.92) | 65275.41 (28775.35-163016.34) | <0.0001 |
| IL-8 levels after Ag2 stimulation, median (IQR) | 20554.28 (1667.37-48377.98) | 74077.24 (39349.67-188186.69) | <0.0001 |
| RANTES non stimulated, median (IQR) | 18605.75 (9804.38-43057.46) | 19774.63 (10986.93-39209.84) | 0.534 |
| RANTES levels after Ag1 stimulation, median (IQR) | 22691.1 (12675.34-52699) | 25220.33 (14131.33-57622.12) | 0.696 |
| RANTES levels after Ag2 stimulation, median (IQR) | 22608.19 (11366.92-54791.4) | 22749.58 (9824.11-54437.42) | 0.851 |
| CD40L/CD137/CD4, median (IQR) | 0.0319 (0.00-0.093) | 0.032 (0.000627-0.296) | 0.478 |
| CD40L/IFN-g/CD4, median (IQR) | 0.00342 (000-0.024) | 0.0078 (0.000-0.074) | 0.055 |
| CD40L/IL-2/CD4, median (IQR) | 0.011 (0.00-0.08) | 0.01659 (0.00-0.277) | 0.377 |
| CD40L/IL-17/CD4, median (IQR) | 0.017 (0.00-0.0734) | 0.030 (0.00007-0.277) | 0.217 |
| CD40L/CD137/CD8, median (IQR) | 0.00086 (0.000-0.031) | 0.000035 (0.0000-0.07) | 0.675 |
| CD40L/IFN-g/CD8, median (IQR) | 0.0000 (0.000-0.016) | 0.0000 (0.0000-0.0072) | 0.965 |
| CD40L/IL-2/CD8, median (IQR) | 0.000 (0.000-0.032) | 0.000 (0.000-0.066) | 0.478 |
| CD40L/IL-17/CD8, median (IQR) | 0.000429 (0.000-0.033) | 0.0000 (0.000-0.071) | 0.478 |

ABA: abatacept

HC: healthy control

IFN-γ: interferon-γ

IQR: interquartile range

R²: R-squared for each dependent variable
