# Supplementary figures and images for: Efficacy of COVID-19 mRNA vaccination in patients with autoimmune disorders: humoral and cellular immune response
Source: BMC Med. 2023 Jun 14;21:210. doi: 10.1186/s12916-023-02868-w (PMC10266318; doi:10.1186/s12916-023-02868-w)

Supplemental figure 1.


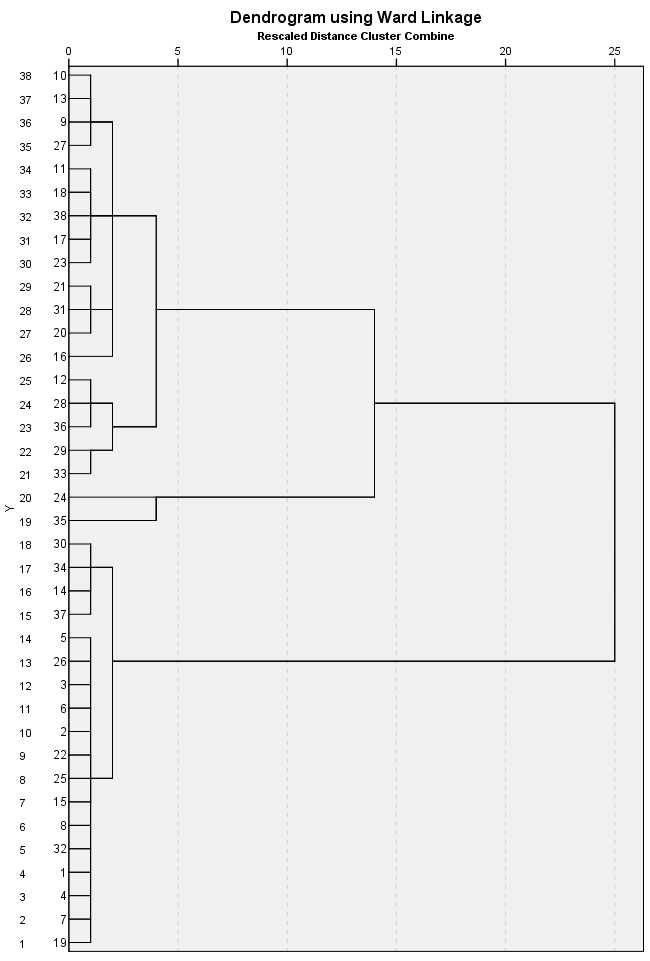

Supplement: Supplementary file 2 — Additional file 2: Supplemental figure 1. Dendogram using Ward Linkage. [file 12916_2023_2868_MOESM2_ESM.docx]

**Supplemental figure 2**


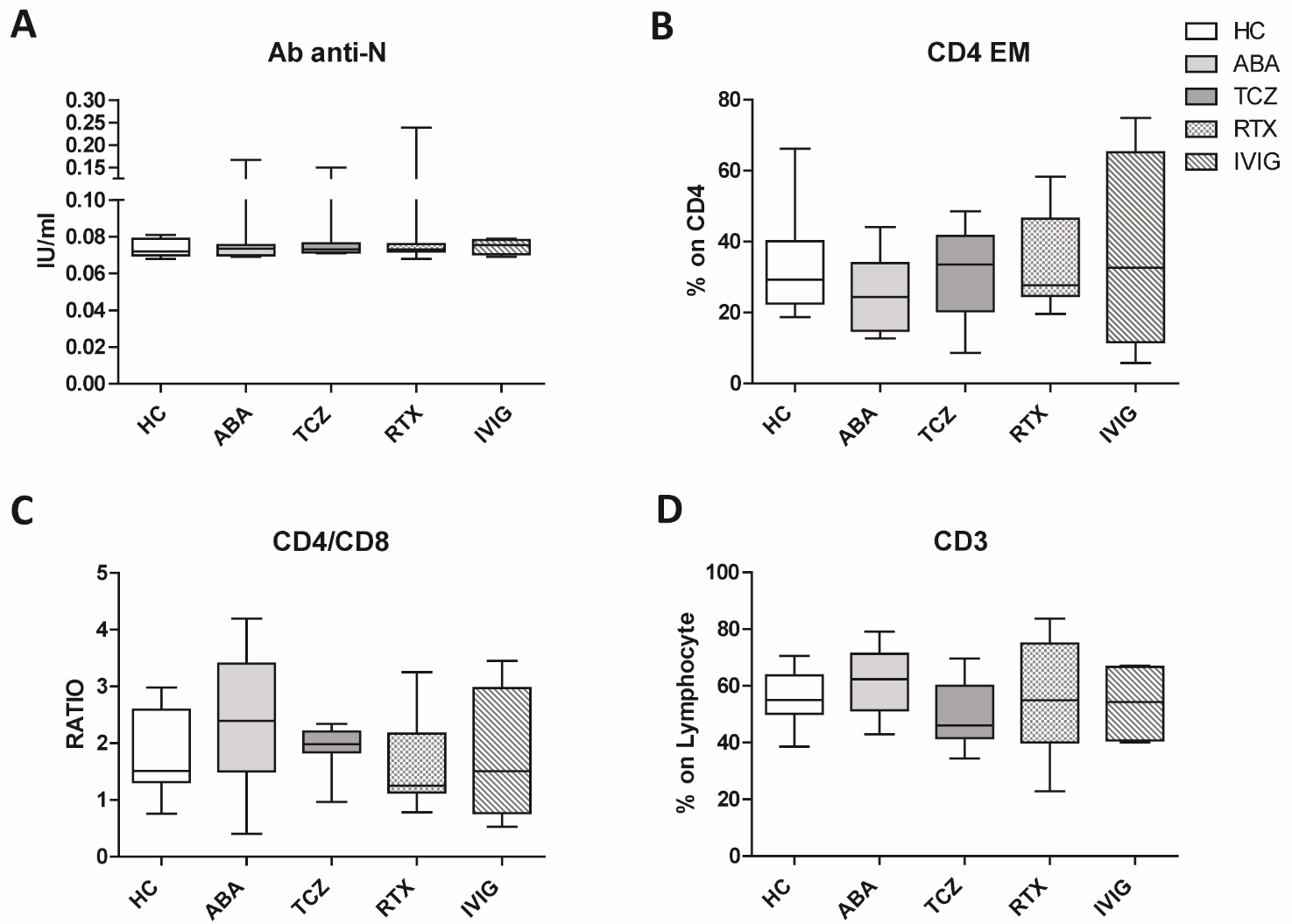

Supplement: Supplementary file 5 — Additional file 5: Supplemental figure 2. Comparison of plasma levels of antibodies anti-nucleocapsid protein (A), effector memory CD4 T cells (B), CD4/CD8 ratio balance (C) and CD3 T cells (D) among the different treatment groups of patients after the second dose of vaccine. The data are shown as box plots. HC=Healthy Controls, ABA=abatacept, TCZ=tocilizumab, RTX=rituximab, IVIG= intravenous immunoglobulin. [file 12916_2023_2868_MOESM5_ESM.docx]

**Supplemental figure 3**


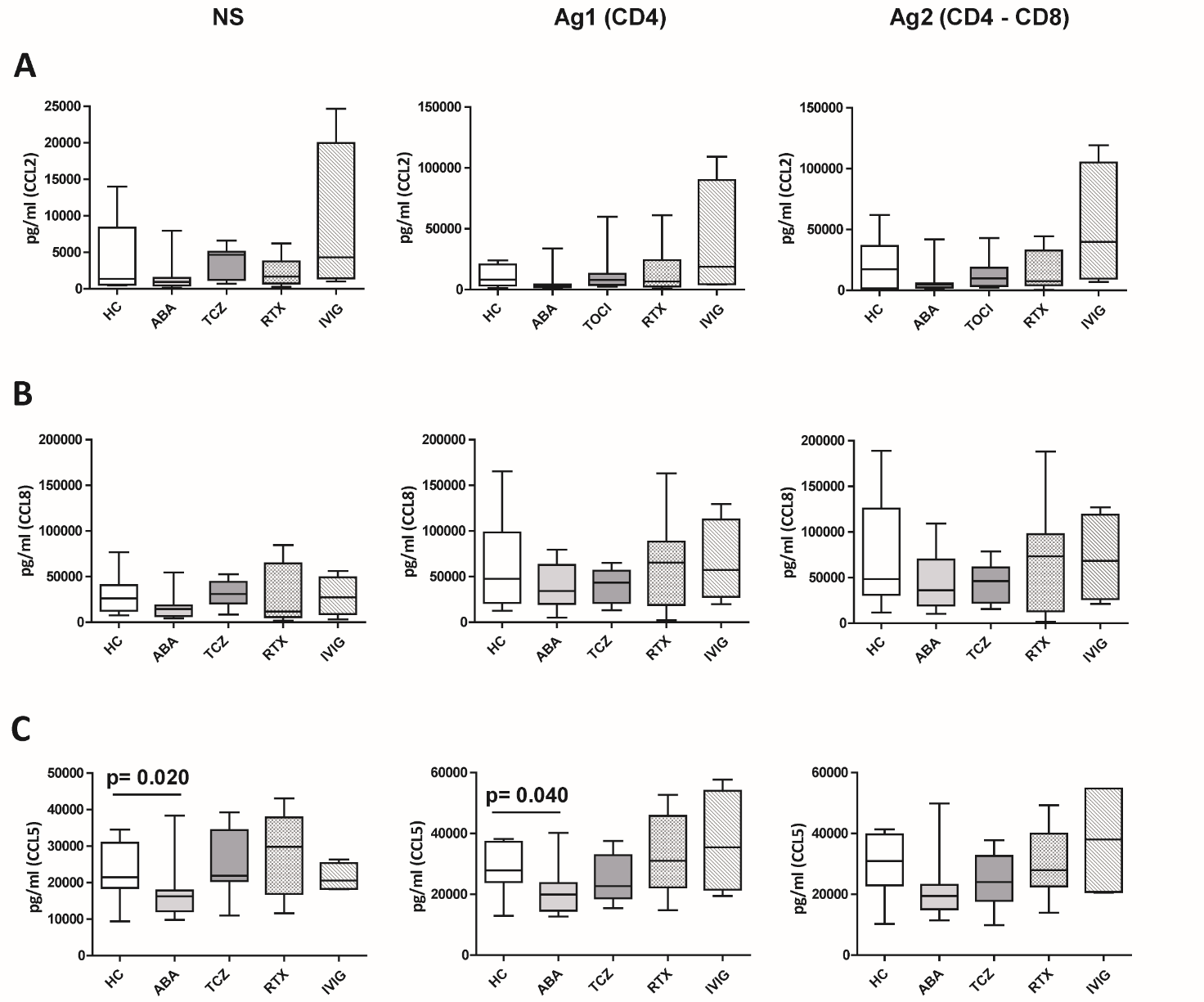

Supplement: Supplementary file 6 — Additional file 6: Supplemental figure 3. Levels of CCL2 (A), CCL8 (B), CCL5 (C) released by unstimulated cells (on the left), CD4 T cells stimulated with spike-derived peptide the different groups of patients, as indicated, after the second dose of COVID-19 vaccine. The data are shown as box plots (extremes of the box are at the bottom the first and at the top the third quartile, the inner row is the median and the upper line and lower line are the highest and lowest values). HC=Healthy Controls, ABA=abatacept, TCZ=tocilizumab, RTX=rituximab, IVIG=intravenous immunoglobulin. [file 12916_2023_2868_MOESM6_ESM.docx]

**Supplemental figure 4**


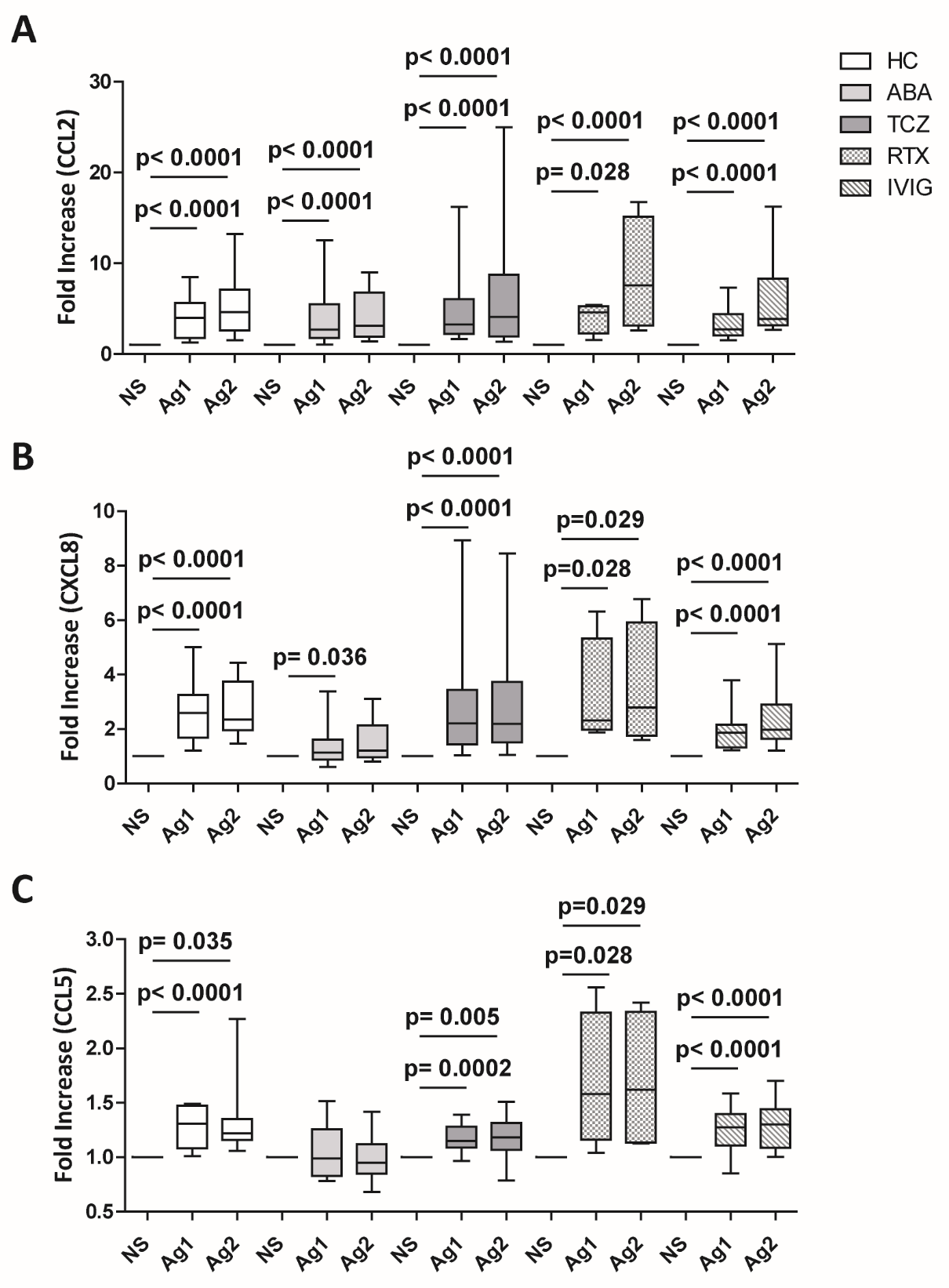

Supplement: Supplementary file 7 — Additional file 7: Supplemental figure 4. Fold increase levels of CCL2 (A), CXCL8 (B) and CCL5 between unstimulated (NS) and CD4 T cells stimulated with spike-derived peptide QFN Ag1, fold increase levels between unstimulated (NS) and CD4/CD8 T cells stimulated with spike-derived peptide QFN Ag2among different groups of patients, as indicated, after the second dose ofCOVID-19 vaccine. The data are shown as box plot (extremes of the box are at the bottom the first and at the top the third quartile, the inner row is the median and the upper line and lower line are the highest and lowest values). HC=Healthy Controls, ABA=abatacept, TCZ=tocilizumab, RTX=rituximab, IVIG=intravenous immunoglobulin. [file 12916_2023_2868_MOESM7_ESM.docx]

**Supplemental figure 5**


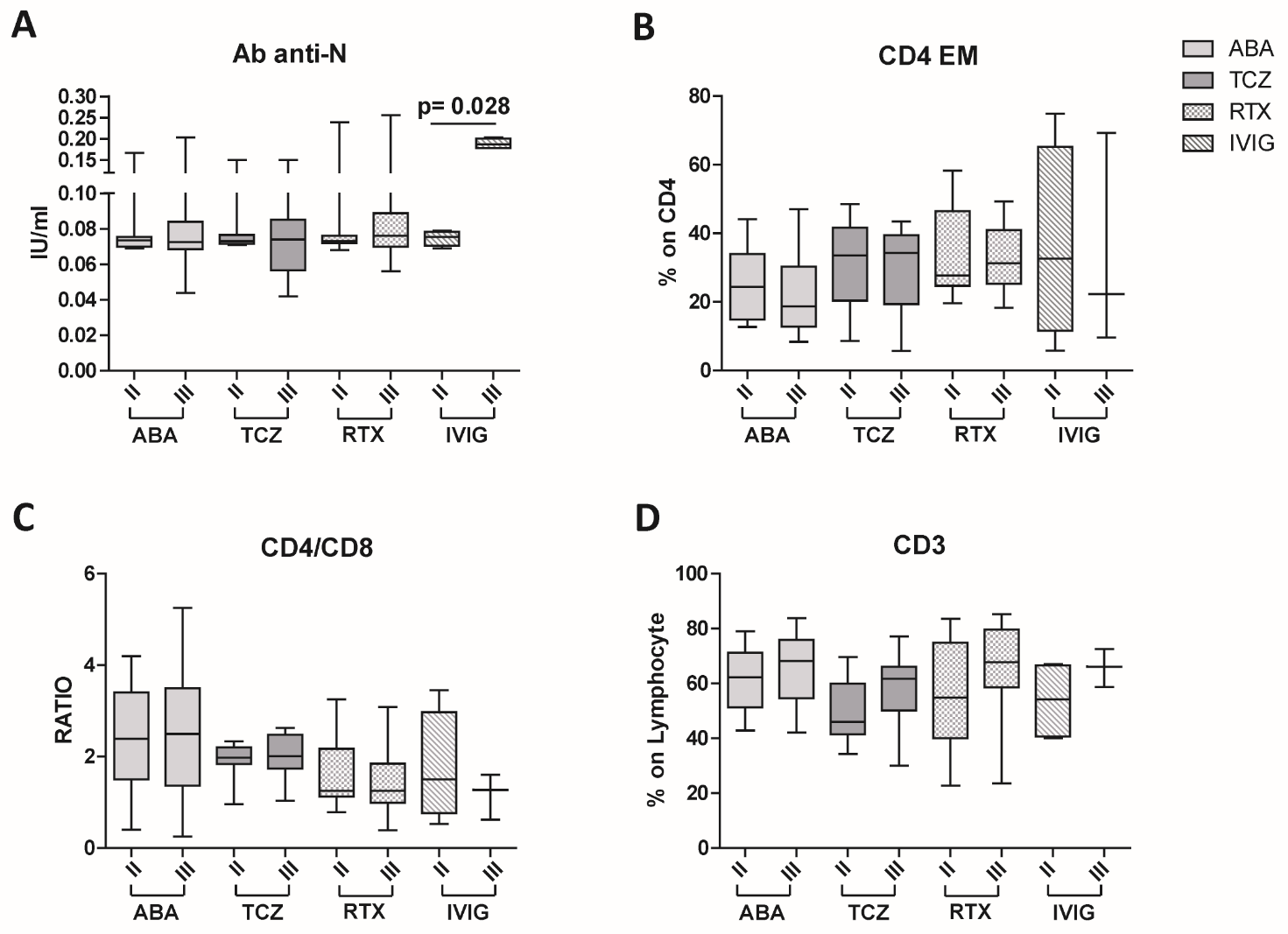

Supplement: Supplementary file 9 — Additional file 9: Supplemental figure 5. Comparison of plasma levels of antibodies anti-nucleocapsid protein (A), effector memory CD4 T cells (B), CD4/CD8 ratio balance (C) and CD3 T cells(D) for each group of patients between the second and third dose of COVID-19 vaccine. The data are shown as box plots (extremes of the box are at the bottom the first and at the top the third quartile, the inner row is the median and the upper line and lower line are the highest and lowest value). HC=Healthy Controls, ABA=abatacept, TCZ=tocilizumab, RTX=rituximab, IVIG=intravenous immunoglobulin. [file 12916_2023_2868_MOESM9_ESM.docx]
