# Supplementary material for: Efficacy of COVID-19 mRNA vaccination in patients with autoimmune disorders: humoral and cellular immune response
Source: BMC Med. 2023 Jun 14;21:210. doi: 10.1186/s12916-023-02868-w (PMC10266318; doi:10.1186/s12916-023-02868-w)
Supplement: Supplementary file 3 — Additional file 3: Supplemental table 2. cluster 1 and 2 composition. [file 12916_2023_2868_MOESM3_ESM.docx]

Supplemental table 2

|  |  | cluster | | Total |
| --- | --- | --- | --- | --- |
|  |  | 1 | 2 |  |
| groups | 1 abatacept | 9 | 5 | 14 |
|  | 2 tocilizumab | 3 | 6 | 9 |
|  | 3 rituximab | 5 | 6 | 11 |
|  | 4 CVID | 1 | 3 | 4 |
| Total | | 18 | 20 | 38 |
