# Supplementary material for: Efficacy of COVID-19 mRNA vaccination in patients with autoimmune disorders: humoral and cellular immune response
Source: BMC Med. 2023 Jun 14;21:210. doi: 10.1186/s12916-023-02868-w (PMC10266318; doi:10.1186/s12916-023-02868-w)
Supplement: Supplementary file 8 — Additional file 8: Supplemental table 3. CXCL10 and CXCL9 fold increase (between Ag1 stimulated and non stimulated cells, and Ag2 stimulated and non stimulated cells) were higher in abatacept groups compared to healthy controls. No significant difference was found in CXCL10 and CXCL9 fold increase between other groups and healthy controls. [file 12916_2023_2868_MOESM8_ESM.doc]

Supplemental table 3: CXCL10 and CXCL9 fold increase (between Ag1 stimulated and non stimulated cells, and Ag2 stimulated and non stimulated cells) in all groups

|  | ABA group  (n.14) | RTX group  (n.10) | TCZ group  (n.10) | CVID group  (n.4) | HC  (n.11) | p* |
| --- | --- | --- | --- | --- | --- | --- |
| Fold increase CXCL10 (Ag1- NS), median (IQR) | 6.33  (1-456.8) | 12.9  (1.53-91.35) | 4.11  (1.11-102.68) | 16.8  (1.75-41.8) | 22.53  (4.08-82.91) | 0.0138 |
| Fold increase CXCL10 (Ag2-NS), median (IQR) | 11.57  (1.44-397.5) | 10.8  (1.47-161.95) | 3.05  (0.9-84.37) | 24.79  (2-48.38) | 35.74  (12.9-153.51) | 0.0073 |
| Fold increase CXCL9 (Ag1-NS), median (IQR) | 2.955  (0.59-42.42) | 5.14  (1.52-132.7) | 1.91  (1-57.28) | 4.47  (1.37-24.67) | 17.03  (2.3-117.23) | 0.01 |
| Fold increase CXCL9 (Ag2-NS), median (IQR) | 4.58  (1.22-40.5) | 5.75  (1.45-314.22) | 1.44  (1.12-79.72) | 6.43  (1.78-29.2) | 32.12  (6.6-160.54) | 0.0044 |

p*: comparison between ABA group and HC

ABA: abatacept

CVID: common variable immunodeficiency

HC: healthy controls

IFN-γ: interferon-γ

IQR: interquartile range

NS: non stimulated

RTX: rituximab

TCZ: tocilizumab
